# The Function of Metformin in Aging-Related Musculoskeletal Disorders

**DOI:** 10.3389/fphar.2022.865524

**Published:** 2022-03-08

**Authors:** Yanhong Song, Ziyi Wu, Ping Zhao

**Affiliations:** Department of Anesthesiology, Shengjing Hospital of China Medical University, Shenyang, China

**Keywords:** metformin, musculoskeletal diseases, aging, drug effects, bone homeostasis

## Abstract

Metformin is a widely accepted first-line hypoglycemic agent in current clinical practice, and it has been applied to the clinic for more than 60 years. Recently, researchers have identified that metformin not only has an efficient capacity to lower glucose but also exerts anti-aging effects by regulating intracellular signaling molecules. With the accelerating aging process and mankind’s desire for a long and healthy life, studies on aging have witnessed an unprecedented boom. Osteoporosis, sarcopenia, degenerative osteoarthropathy, and frailty are age-related diseases of the musculoskeletal system. The decline in motor function is a problem that many elderly people have to face, and in serious cases, they may even fail to self-care, and their quality of life will be seriously reduced. Therefore, exploring potential treatments to effectively prevent or delay the progression of aging-related diseases is essential to promote healthy aging. In this review, we first briefly describe the origin of metformin and the aging of the movement system, and next review the evidence associated with its ability to extend lifespan. Furthermore, we discuss the mechanisms related to the modulation of aging in the musculoskeletal system by metformin, mainly its contribution to bone homeostasis, muscle aging, and joint degeneration. Finally, we analyze the protective benefits of metformin in aging-related diseases of the musculoskeletal system.

## Introduction

Metformin is a widely used oral hypoglycemic agent originally extracted from Galega officinalis, a traditional European herb, and was first reported in 1957 for the treatment of diabetes (Marshall, 2017; Flory and Lipska, 2019). Although it was generally discontinued owing to its reputation being affected by other biguanides (high risk of acidosis), its ability to combat insulin resistance and lower blood sugar has been gradually recognized after intensive research (Bailey, 2017). Metformin has been applied to clinical practice for over 60 years and current perspectives suggest that metformin can not only lower glucose but also serve as a preventive and therapeutic intervention in aging-related diseases such as cardiovascular diseases, neurodegenerative diseases, cancer, and degenerative osteoarthrosis (Nesti and Natali, 2017; Vancura et al., 2018; Soukas et al., 2019; Feng et al., 2020; Lv and Guo, 2020). The protective effect of metformin is largely attributed to its activation of AMPK. It can specifically inhibit complex Ⅰ in the mitochondrial respiratory chain to increase the AMP: ATP/ADP: ATP ratios in the cytoplasm and AMPK is activated as an energy sensor to restore energy balance (Hawley et al., 2010; Foretz et al., 2014; Rena et al., 2017). The emerging insights suggest that the activation of AMPK is not only the result of disruption of ATP metabolism through oxidative phosphorylation. In hepatocytes, the scaffolding protein (AXIN) also plays an essential role in the activation of AMPK by co-transporting LKB1 (AMPK upstream kinase) to the lysosomal surface (Zhang et al., 2016).

The movement system is comprised of three main parts: bone, joints, and skeletal muscle. Bone tissue is the connective tissue that is composed of cells, fibers, and matrices. Organic matter and inorganic salts together constitute the bone matrix, and a large amount of deposited inorganic salts characterizes the bone tissue as hard. However, as we age, the proportion of inorganic components gradually increases, so bone tissue in elderly patients is characterized by hard and brittle (Burr, 2019). Age-related aging of the skeletal system is a result of the combined effects of multiple organ systems. Changes in hormone levels and sensitivity of organs to hormones, decreased mobility, reduced nutrient absorption capacity, complex comorbidities, and long-term oral hormonal medications all combine to disrupt bone homeostasis, accelerate bone resorption, and slow bone formation in the elderly, ultimately causing bone loss (Curtis et al., 2015). Joint aging is a physiological condition that most older adults will experience. Cartilage degeneration and osteoporosis cause a significant decrease in joint mobility. Wear and tear of joints due to excessive exercise, metabolic dysfunction due to obesity, lack of exercise, and uneven stress on joints for a long period are all risk factors for joint aging (Palazzo et al., 2016). Skeletal muscle also undergoes structural and functional changes with advancing age, usually in the form of decreased muscle mass, strength, and regenerative capacity (Aoyagi and Shephard, 1992; Faulkner et al., 2007; Distefano and Goodpaster, 2018)**.** Muscle atrophy is the result of a combination of mitochondrial changes (Leduc-Gaudet et al., 2015; Fukunaga et al., 2021), chronic inflammation (Tuttle et al., 2020), insulin resistance (Keske et al., 2016), and diminished muscle regeneration (Verdijk et al., 2007; Sousa-Victor et al., 2014). The aging of the motor system is closely associated with falls, fractures, and decreased quality of life in the elderly. Aging is a natural law for the development of human organism function, but the effective delay of aging and promotion of healthy aging are the goals of anti-aging research.

## Metformin and Life Extension

Experimental animal studies have shown that metformin can extend the lifespan of nematodes and rodents. Host and microorganisms can interact physiologically, and C.*elegans* under co-culture conditions with *E. coli*, metformin leads to altered nematode metabolism and increased longevity by regulating folate and methionine metabolism in *E. coli* (Cabreiro et al., 2013). Another study also demonstrates that metformin can prolong the average lifespan of female mice and that this anti-aging effect is age-dependent. Using pharmacological interventions at a younger age to extend lifespan appears to be more effective (Anisimov et al., 2011). In addition, male mice with chronic dietary intake of low doses of metformin lived longer compared to controls, but intake of high concentrations of metformin instead resulted in a shorter lifespan, which may be associated with lactic acidosis and renal insufficiency as side effects of metformin (Martin-Montalvo et al., 2013). Experiments in adult *Drosophila* did not yield consistent findings. Metformin did activate AMPK and reduce fat accumulation in *Drosophila*, but this did not lead to increased lifespan (Slack et al., 2012). However, metformin is still a drug with the potential to extend lifespan, and various factors such as species variability, drug concentration, and age of the subject may affect the anti-aging effects of metformin.

A retrospective study involving approximately 180,000 people found that patients with type 2 diabetes treated with metformin monotherapy had a 15% longer median survival compared to matched non-diabetic patients (Bannister et al., 2014). Since this is a retrospective observational study, the findings may be subject to some confounding factors, but this does not affect the potential of metformin as an anti-aging drug. In 2015, the United States Food and Drug Administration (FDA) approved the first clinical trial of metformin in humans for anti-aging (Targeting Ageing with Metformin). The trial plans to enroll 3,000 non-diabetic individuals at risk for aging-related disease and take 1,500 mg of metformin daily for 6 years, with the endpoint of the study being the development of aging-related disease (including coronary heart disease, stroke, congestive heart failure, peripheral artery disease, cancer, type 2 diabetes (T2D), cognitive impairment, etc.) (Barzilai et al., 2016), and the results of the trial have not yet been published.

## Mechanisms of Metformin Regulation of the Musculoskeletal System

### Metformin and Bone

#### Inducing Differentiation of Mesenchymal Stem Cells to Osteoblasts

**FIGURE 1:**
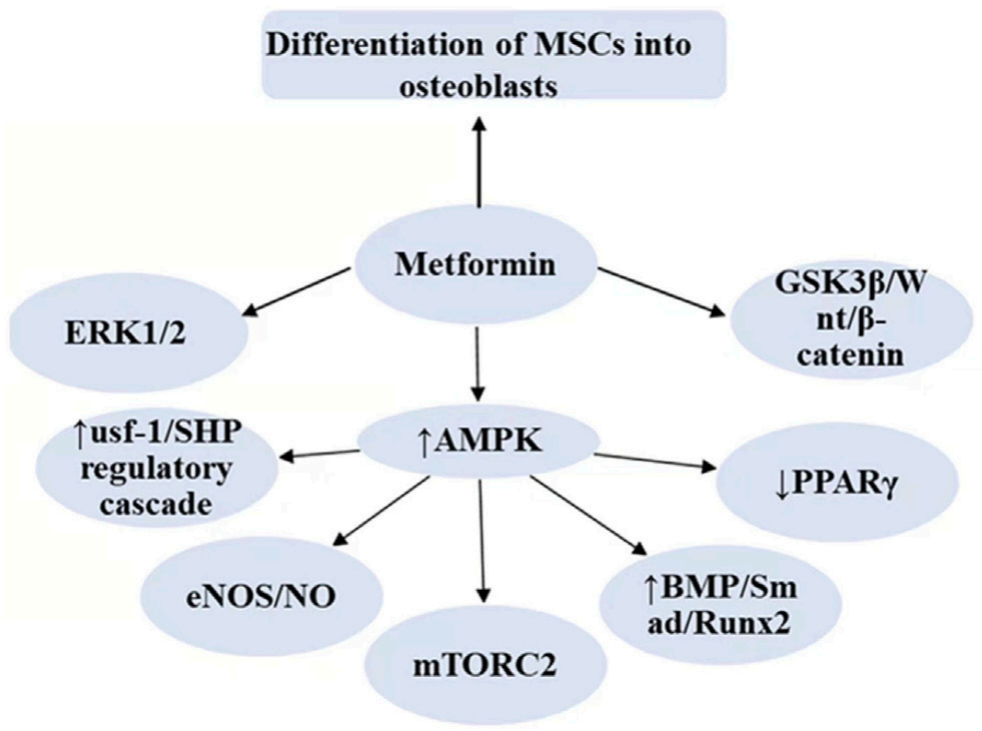
The effects of metformin in the differentiation of MSCs to osteoblasts.

Mesenchymal stem cells (MSCs) are pluripotent stem cells with the ability of self-renewal and multidirectional differentiation, which can be directed to different cell types under different induction conditions. Genes such as Runx2, alkaline phosphatase (ALP), and osteopontin (OPN) are usually regarded as osteogenic markers, and they play a critical position in the osteogenic differentiation of MSCs (Vimalraj et al., 2015). Several studies have revealed that metformin has a role in promoting osteoblastogenesis in MSCs differentiation (Cortizo et al., 2006; Gao et al., 2008; Gu et al., 2017; Lei et al., 2021). Metformin regulates the differentiation of MSCs to osteoblasts probably mediated by AMPK, bone morphogenetic proteins (BMPs), glycogen synthase kinase-3β(GSK3β), AKT, and ERK (As shown in Figure 1). In an *in vitro* culture of induced pluripotent stem cell-derived MSCs, metformin treatment upregulated AMPK expression and promoted osteogenic differentiation of MSCs, while inhibition of LKB1 activity reversed metformin-stimulated AMPK activation and decreased the expression of osteogenic markers (e.g., Runx2) (Wang et al., 2018). The connections between intracellular molecules are complex and intertwined, and the osteogenic role of AMPK as a sensor of cellular energy homeostasis and metabolic homeostasis may be achieved through multiple pathways. Activated AMPK may play a role in the regulation of bone differentiation by activating downstream signaling molecules, such as usf-1, endothelial nitric oxide synthase (eNOS), mTORC, and BMPs. It has been shown that low concentrations of metformin stimulate the differentiation of mouse cranial-derived cells into osteoblasts via promoting the trans-activation of runx2 by AMPK/usf-1/SHP regulatory cascade (Jang et al., 2011). Emerging insights suggest that the AMPK/eNOS/NO (nitric oxide) pathway may also be involved in the osteogenic effects of metformin. The effect of metformin on osteogenesis and adipocyte differentiation of CV-MSCs (human villous mesenchymal stem cells) was investigated. Metformin (0.05 mM) induced endothelial nitric oxide synthase and thus promoted osteogenesis, whereas PPARγ (peroxisome proliferator-activated receptor γ), which is associated with adipocyte synthesis, was significantly inhibited at late stages of differentiation (Gu et al., 2017). The AMPK-mTORC2 signaling pathway is involved in the promotion of the proliferation of mouse preosteoblasts by metformin (Mu et al., 2018). In addition, BMPs, which are members of the transforming growth factor-β family, play a role in promoting osteogenic differentiation by stimulating DNA synthesis and cell replication. Metformin can play a pro-osteoclastogenic role by activating AMPK/BMP/Smad signaling pathway (Sun et al., 2021) Another similar study also confirms that metformin promotes the osteogenic function of pro-osteoblasts in type 2 diabetic patients through the BMP-4/Smad/Runx2 axis (Liang et al., 2020). GSK3β can also act as a mediator in the process of metformin-promoted osteoblast differentiation. Metformin activates the Wnt/β-catenin signaling pathway in human mesenchymal stem cells by inhibiting the activity of GSK-3β, which ultimately increases the accumulation of β-catenin in the nucleus (Ma et al., 2018). The Wnt/β-catenin signaling pathway has been shown to play a critical role in the osteogenic differentiation of MSCs. When Wnt is activated, β-catenin in the cytoplasm is transported to the nucleus where it binds to transcription factors and regulates the expression of related genes downstream (Baron and Kneissel, 2013; Shen et al., 2020). AKT is a protein kinase associated with cell survival and apoptosis, and *in vitro* experiments suggest that metformin reverses oxidative stress damage induced by a high glucose environment through the ROS-AKT-mTOR axis and promotes the differentiation of bone marrow mesenchymal stem cells into osteoblasts (Zhou et al., 2020). In another experiment, AKT-mTORC1 is also found to be involved in the proliferation of pro-osteoblasts (Mu et al., 2018). Activation and redistribution of ERK-1/2 also play a role in metformin-promoted osteoblast differentiation (Cortizo et al., 2006). In conclusion, in the molecular mechanism of metformin-promoted osteoblastogenesis, each signaling molecule does not exist independently, and there are complex interactions among them, but what is clear is that a large number of experiments have confirmed the ability of metformin to promote the conversion of mesenchymal stem cells to osteoblasts.

#### Impact on Osteoblast Function

Osteoblasts are key cells in bone formation that promote the synthesis, secretion, and mineralization of the bone matrix. The non-collagenous components of the organic matrix (amorphous matrix) usually contain alkaline phosphatase, osteocalcin, osteonectin, cellular ligand proteins, and skeleton growth factors. These amorphous matrices play a key role in the maturation and mineralization of the extracellular matrix (Dirckx et al., 2019). In an investigation of osteoblast cell lines cultured *in vitro*, Cortizo et al. (2006) demonstrated for the first time that metformin had a role in promoting osteoblast bone formation, and that metformin treatment not only promoted the proliferation of osteoblast cell lines, upregulated type I collagen expression and alkaline phosphatase activity, but also stimulated the formation of cell mineralized nodules. Another study further determined that metformin enhanced osteoblast differentiation and mineralization by activating AMPK, inducing eNOS and BMP-2 expression (Kanazawa et al., 2008). Conversely, Kasai et al. discovered that metformin inhibited the expression of the osteoblast Runx2 gene, osteocalcin, osteopontin, and other markers of osteoblast differentiation (Kasai et al., 2009). In contrast to the findings of previous investigators, the source of the difference may be the difference in metformin concentration. The vast majority of current experiments support a protective effect of metformin on osteoblasts. By adding H_2_O_2_ to MC3T3-E1 cells to induce oxidative stress injury model, the experimental results suggested that metformin could repair mitochondrial damage and inhibit osteoblast apoptosis by activating PI3K/AKT signaling pathway to upregulate SIRT3 expression (Yang et al., 2021). Similarly, another study found that metformin promoted the secretion of BMP-2, ALP, and OCN in the bone tissue of diabetic rats, and its protective mechanism may be achieved by inhibiting the TLR4/MyD88/NF-κB signaling pathway (Zheng et al., 2019). Conclusively, metformin may have a potential role in promoting osteoblast proliferation, differentiation, and mineralization.

#### Impact on Osteoclast Function

In the regulation of bone metabolism, activated osteoclasts promote matrix lysis and the emigration of calcium from bone, a process also known as bone resorption, and osteoclasts are key leaders in bone resorption. It is generally accepted that the process of osteoclast generation is as follows: embryonic erythro-myeloid progenitors proliferate and diffuse into monocytes, and the circulating monocytes enter the tissue and transform into macrophages, which eventually fuse to form multinucleated osteoclasts (Jacome-Galarza et al., 2019). Differentiation from osteoclast precursors into osteoclasts is dependent on the binding of the NF-κB receptor activator ligand RANKL to RANK (Park et al., 2017). RANK receives activation signals and activates downstream signaling molecules such as mitogen-activated protein kinases (MAPKs), transcription factor nuclear factor-κB (NF-κB), activator protein-1 (AP-1), activated T cell cytoplasm 1 (NFATc1), and c-Fos through tumor necrosis factor receptor-associated factor 6 (TRAF6) and calcium channels, thereby promoting the generation of osteoblasts. NFATc1, a key regulator of osteoclastogenesis, can translocate to the nucleus to activate target genes associated with osteoclastogenesis (Park et al., 2017; Salminen et al., 2019; Tong et al., 2020).

Many studies have shown that metformin, as a potential AMPK activator, can inhibit osteoclast production and function (Lee et al., 2010; Mai et al., 2011; Park et al., 2020). In mice, bone marrow mesenchymal stem cells (BMSCs) treated with 50 mM metformin showed a significant inhibition of osteoclastogenesis accompanied by a reduction in the area of resorption traps in bone fragments (Lee et al., 2010). Metformin can increase OPG/RANKL ratio in osteoblasts by activating AMPK, which in turn reduces RANKL/RANK binding (Mai et al., 2011). In addition, metformin can also down-regulate the levels of osteoclastogenesis-related signaling molecules. Activated AMPK can down-regulate C-fos, NFATc1 and NF-κB levels (Oh et al., 2016; Salminen et al., 2019; Zhao et al., 2020). Both TNF-α and IL-6 (downstream of NF-κB) can positively regulate osteoclast differentiation and function (Luo et al., 2018; Wang and He, 2020). In contrast, inhibition of AMPK activation by using compound C activated the expression of downstream signals (p38, JNK, NF-κB, Akt, CREB, c-Fos, and NFATc1), ultimately promoting RANKL-induced osteoclast formation (Lee et al., 2010). The α subunit is the catalytic subunit of AMPK and has two isoforms, α1 and α2. AMPKα1 knockdown enhanced RANKL-induced phosphorylation of downstream signaling components. Whereas, in an experimental model with AMPKα2 deletion, there was no significant change in known signaling pathways downstream of RANKL. AMPKα1 appeared to negatively regulate RANKL signaling and had a stronger inhibitory effect on osteoclastogenesis than α2 (Kang et al., 2013). Whereas AMPKα2-silencing was associated with reduced expression of osteogenic markers (mRNA and protein levels of OPG, OCN, OPN, ALP, and BMP6 and protein expression of p-Smad5/Smad5) (Wang et al., 2016; Fan et al., 2017), AMPKα2 overexpression appeared to have a more potent osteogenic effect.

#### Protection of Bone Cells From Damage Caused by Hyperglycemia-Related Metabolites

Diabetes is a disease characterized by increased blood sugar and metabolic disorders. People with diabetes have a significantly increased risk of fractures (Kurra et al., 2014; Shanbhogue et al., 2016; Compston, 2018). Bone damage caused by hyperglycemia may not only be a function of blood glucose itself, but the metabolic disorders triggered by elevated blood glucose may cause adverse effects on bone metabolism. Advanced glycosylation end products (AGEs), succinate in the tricarboxylic acid cycle may also be risk factors for bone damage (Franke et al., 2007; Franke et al., 2011; Guo et al., 2017). Hyperglycemia not only decreases the levels of osteogenic markers (ALP, OCN, OPG, and RUNX2) (Zhou et al., 2020) but also inhibits the growth-promoting effects of several growth factors, including insulin-like growth factor-1, on osteoblasts (Jiating et al., 2019). AGEs are covalent compounds produced by non-enzymatic glycosylation reactions (also known as Maillard reactions) using amino groups of proteins, fats, and nucleic acids and aldehyde groups of reducing sugars as raw materials (Perrone et al., 2020). Accumulation of AGEs not only inhibits osteoblast growth and extracellular matrix mineralization but also promotes osteoclastogenesis, which plays a role in the development of osteoporosis (Franke et al., 2007; Franke et al., 2011). The accumulation of AGEs upregulates the expression of RAGE, a receptor for late glycosylation end products in bone marrow progenitor cells (BMPC) (Tolosa et al., 2013). Studies on bone marrow-derived macrophages in mice found that metformin inhibited the inflammatory response triggered by AGEs, mainly through activation of AMPK and inhibition of the RAGE/NF-κB pathway (Zhou et al., 2016). Another study in osteoblasts came to a similar conclusion that metformin inhibited AGEs-induced apoptosis in osteoblasts, possibly by reversing the AGEs-induced upregulation of RAGE expression (Schurman et al., 2008). The tricarboxylic acid (TCA) cycle is one of the metabolic pathways damaged by hyperglycemia, and succinate, an important intermediate product of TAC, accumulates abnormally under hyperglycemia induction. Succinate has been found to promote osteoclastogenesis and bone resorption *in vivo* and *in vitro*. Treatment with metformin reduced osteoclastogenesis by approximately 20%, probably due to the ability of metformin to antagonize succinate on NF-κB signaling and thus exert osteoprotective effects (Guo et al., 2017).

### Metformin and Articular Chondrocytes

**FIGURE 2:**
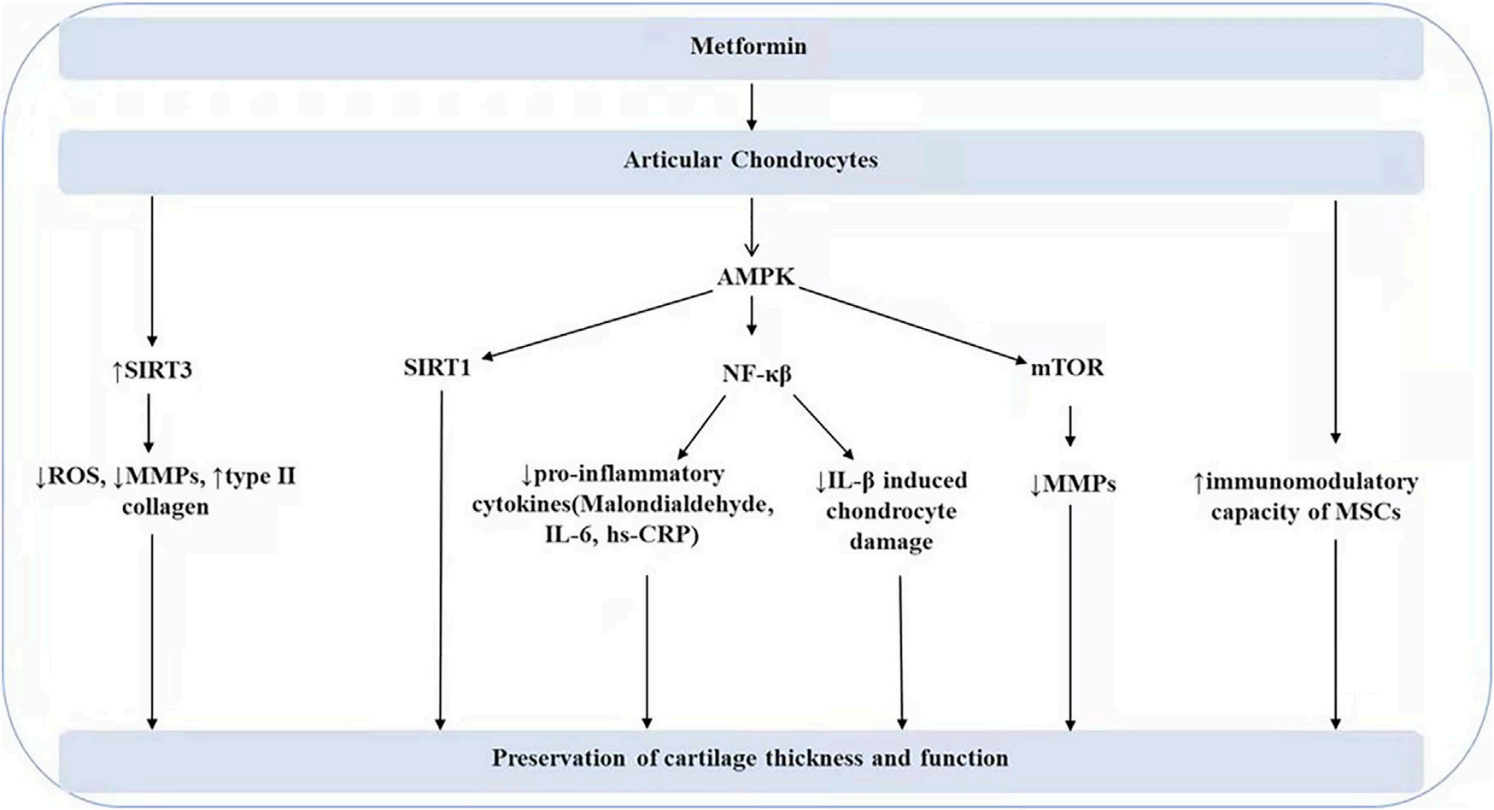
The effect of metformin on articular chondrocytes.

Articular cartilage is attached to the bone in the joint and is responsible for cushioning the bone from shock and impact during movement. However, as we age, articular cartilage degeneration and osteophytes cause a significant decrease in joint mobility. The thinning cartilage tends to break down and form free bodies, causing joint pain. Senescent cells promote the expression of matrix metalloproteins (MMPs), which in turn lead to degradation of the extracellular matrix, the stability of which is essential for the protection of articular cartilage function (Rahmati et al., 2017). Studies have shown that metformin inhibits oxidative stress and cellular inflammation and exerts a protective effect against matrix degradation and chondrocyte apoptosis (Fan et al., 2020; Schadler et al., 2021). AMPK activity in chondrocytes declines with aging (Petursson et al., 2013; Zhou et al., 2017), and AMPK α1 deficiency accelerates the progression of cartilage degeneration (Zhou et al., 2017; Li et al., 2020). Metformin acts as an AMPK activator, which inhibits NF-κB expression, reduces the release of pro-inflammatory cytokines, and blocks IL-β-induced chondrocyte damage (Zhang et al., 2020). Malondialdehyde, interleukin 6, and high-sensitivity C-reactive protein (hs-CRP) levels are significantly reduced by metformin treatment, and there is a significant correlation between these biomarkers of oxidative stress and inflammation and cartilage thickness and function (Dawood et al., 2020). Matrix metalloproteins are the main enzymes that promote the degradation of the extracellular matrix. Metformin concentration is negatively correlated with matrix metalloprotein expression levels, which may be related to the regulation of the AMPK/mTOR signaling pathway (Feng et al., 2020). Another study has found that activation of AMPKα/SIRT1 signaling is also a pathway through which metformin exerts its chondroprotective effects (Wang et al., 2020). Furthermore, sirt3 signaling molecules also Mediate the protective effects of metformin by upregulating sirt3 expression, with decreased production of reactive oxygen species ROS in mitochondria, downregulation of matrix catabolic protein gene expression, and increased type II collagen synthesis (Chenzhong Wang et al., 2019). In summary, existing studies have found that metformin plays a protective role in chondrocyte injury by regulating AMPK/NF-κB, AMPK/mTOR, AMPK/sirt1, and SIRT3 signaling molecules that inhibit inflammation, antagonize oxidative stress, and regulate mitochondrial autophagy (As shown in Figure 2).

### Metformin and Skeletal Muscle Aging

**FIGURE 3:**
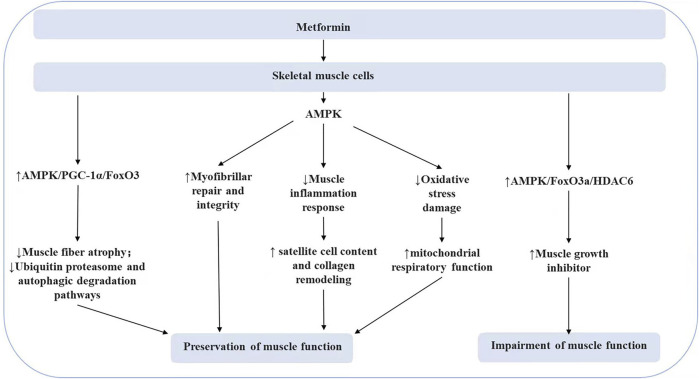
The effect of metformin on skeletal muscle cells.

PGC-1α can exert an essential regulatory role in skeletal muscle aging, atrophy, and functional recovery (Petrocelli and Drummond, 2020). Activated PGC-1α can downregulate FoxO3 to prevent muscle atrophy (Sandri et al., 2006), inhibit the ubiquitin-proteasome system, and autophagic degradation pathways to protect muscle function (Cannavino et al., 2014). Metformin has been demonstrated to promote the expression of PGC-1α in skeletal muscle through the activation of AMPK (Suwa et al., 2006). Metformin treatment was found to improve myofiber atrophy, fibrosis, and increased E3 ubiquitin ligase expression induced during a high-fat diet, and this protective effect was partially attributed to the regulation of the PGC-1α/FoxO3 signaling pathway by metformin (Hasan et al., 2019). Metformin can also modulate satellite cells in muscle. The combination of metformin and leucine can positively affect aging muscle function by increasing satellite cell content and modulating collagen remodeling (Petrocelli et al., 2021). Moreover, metformin can attenuate the muscle inflammatory response in diabetic patients (Peixoto et al., 2017), promote the repair of myofilament damage in skeletal muscle fibers (Ono et al., 2020; Dong et al., 2021), and protect the respiratory capacity of skeletal muscle by counteracting oxidative stress damage (Kane et al., 2010). However, Stockinger et al. (2017) discovered that metformin did not significantly affect the aging of the neuromuscular junction. Exercise training is an effective measure to inhibit muscle atrophy. A study found that in patients with prodromal diabetes, metformin use attenuated the muscle-protective effects of exercise training (Malin et al., 2012; Walton et al., 2019), possibly attributed to metformin limiting exercise-mediated increases in mitochondrial respiration in skeletal muscle (Konopka et al., 2019). Additionally, it was proposed that metformin could inhibit muscle hypertrophy by modulating the AMPK/mTORC1 signaling pathway to reduce muscle protein synthesis or increase autophagy (Kwon and Querfurth, 2015). But the inhibition of mitochondrial function may be caused by high metformin concentration (≥1 mM) (Stockinger et al., 2017; Petrocelli and Drummond, 2020). Metformin also promotes muscle growth inhibitory factor release via the AMPK/FoxO3a/HDAC6 axis and even impairs muscle function (As shown in Figure 3). In summary, the concentration and dose of metformin, species differences in experimental subjects, incubation conditions, and metabolic status may have an impact on the experimental results, and the role of metformin in skeletal muscle aging remains to be further explored.

## Protective Effects of Metformin in the Musculoskeletal System

Metformin is a promising anti-aging drug involved in the regulation of bone homeostasis, muscle aging, and joint degeneration through the modulation of intracellular signaling. Osteoporosis, sarcopenia, degenerative osteoarthropathy, and frailty are all diseases closely associated with the aging of the locomotor system. Next, we summarize the role of metformin in the pathology of the musculoskeletal system (Table1). Species variability, drug dose, and method of administration may lead to differences in experimental findings, but the anti-aging potential of metformin deserves further exploration.

**TABLE 1:** Summary of the role of metformin in the musculoskeletal system.

disease	Metformin dosage	Models	Experimental subjects	References	Public year	Description
Osteoporosis	0.05–0.4 mM *in vitro*, and 100 mg/kg/day by intragastric injection	H2O2 added to MC3T3-E1 cells and ovariectomy-induced osteoporosis in a mouse model	MC3T3-E1 cells and C57BL/6J female mice	Yang et al. (2021)	2021	Inhibited oxidative stress and osteoblast apoptosis by regulating PI3K/AKT/SIRT3 pathway
	50 mM *in vitro* and submucosally injected with 20 mg/ml metformin *in vivo*	Ovariectomy-induced osteoporosis in a rat model	12-week-old female Sprague-Dawley rats	Lin et al. (2020)	2020	Promoted the osseointegration through regulating BMSCs autophagy, and osteogenic differentiation
	100 mg/kg/day by orogastric intubation	Ovariectomy-induced osteoporosis	adult Sprague–Dawley female rats	Mai et al. (2011)	2011	Increased OPG/RANKL ratio and thereby reduce osteoclast differentiation and bone loss
	200 mg/kg/day by gavage	Glucocorticoid (GC)-induced osteoporosis in a rat model	3-month-old female Sprague–Dawley rats	Zhao et al. (2019)	2019	Prevented bone loss by suppressing bone resorption and promoting bone formation
	100 mg/kg by intraperitoneal injection	Ketogenic diet-induced osteoporosis in mice model	6-week-old female C57BL/6J mice	Liu et al. (2019)	2019	Enhanced osteoblast proliferation and inhibited osteoclast differentiation by Increasing the expression of ALP and OCN, downregulating TRAP expression
	250 mg/kg by oral administration	In an osteoporosis rat model administered zoledronic acid (ZA) and dexamethasone (DX)	male Wistar rats	Nakagawa et al. (2021)	2021	Prevented bisphosphonate-related osteonecrosis
	Administration of metformin	In Taiwanese patients with type 2 diabetes mellitus	Human	Tseng, (2021)	2021	Reduced the risk of both osteoporosis and vertebral fracture by 30–40%
	Administration of metformin	High risk of diabetes (mean age was 66.5 ± 9.5 years)	Human	Schwartz et al. (2021)	2021	Femoral neck bone mineral density was higher in the metformin compared to the placebo group
	Administration of metformin	Overweight patients with type 2 diabetes	Human	Nordklint et al. (2021)	2021	Metformin treatment has a small but positive effect on the mineral content and density of peripheral bone
	Administration of metformin	Latin American adult women	Human	Blümel et al. (2020)	2020	lower risk of osteoporosis in adult women regardless of the presence of type 2 diabetes or obesity
Sarcopenia	Administration of metformin	In patients with T2DM	Human	Ai et al. (2021)	2021	Reduced risk of sarcopenia in patients with type 2 diabetes
	400 μM *in vitro*, and 250 mg/kg/day by adding to the drinking water	Sedentary 12-week-old C57BL/6 mice	Mouse C2C12 myoblasts and 12-week-old Male C57BL/6 mice	Senesi et al. (2016)	2016	Anti-oxidative stress; promoted skeletal muscle differentiation and myotubular maturation by regulating signaling molecules such as ERKs and AKT; prevented sedentariness damages
	250 mg/kg by intraperitoneal injection three times a week	Three-month-old and 23-month-old mice were euthanized to obtain the tibialis anterior muscle tissue	C2C12 myoblasts and male C57BLKS/J-db/db and C57BL/6J mice	Kang et al. (2021)	2021	Impaired muscle function by modulating myostatin in skeletal muscle cells via the AMPK-FoxO3a-HDAC6 pathway
	100 mg/kg by oral administration	Sedentary/exercise rats	Wistar female rats	Hernández-Álvarez et al. (2019)	2019	The combination of exercise and metformin prevented strength and muscle mass loss
	Administration of 1,700 mg/d	Progressive resistance exercise training in older adults	Human	Walton et al. (2019)	2019	Inhibited the hypertrophic response to resistance training
Degenerative Osteoarthropathy	1–5 mM *in vitro*, and 100 mg/kg/d or 200 mg/kg/d by oral gavage	Destabilization of the medial meniscus (DMM) surgery-induced osteoarthritis model and Primary articular chondrocytes	8-week-old male C57BL/6 mice	Feng et al. (2020)	2020	Attenuated cartilage degeneration by regulating AMPK/mTOR
	205 mg/kg by dissolving in drinking water (7 times a week)	Destabilization of the medial meniscus (DMM) surgery-induced osteoarthritis model	AMPKα1 KO mice and congenic wild-type (WT) mice with C57BL/6/129 background	Li et al. (2020)	2020	Delayed development and progression of osteoarthritis by activating AMPK
	Stimulated Ad-hMSCs with metformin (1 mM) for 48 h	Intra-articular injection of monosodium iodoacetate induced osteoarthritis model	Six-week-old male Wistar rats	Park et al. (2019)	2019	Enhanced the immunomodulatory capacity of mesenchymal stem cells (MSCs), leading to greater analgesic activity and chondroprotective effects
	Combination therapy with COX-2 inhibitors and metformin	In osteoarthritis patients with T2DM	Human	Lu et al. (2018)	2018	Reduced rate of joint replacement surgery
	Administration of metformin	In obese people with knee osteoarthritis	Human	Yuanyuan Wang et al. (2019)	2019	Reduced rate of medial cartilage volume loss and risk of total knee arthroplasty
Frailty	Administration of metformin	veterans 65 years and older with type 2 diabetes	Human	Baskaran et al. (2020)	2020	Reduced risk of frailty syndrome
	Administration of metformin	Elderly people with type 2 diabetes (≥60 years old)	Human	Sumantri et al. (2014)	2014	Reduced risk of frailty syndrome
	Administration of metformin	Participants in the Diabetes Prevention Program (DPP) clinical trial	Human	Hazuda et al. (2021)	2021	Potentially ineffective in reducing the prevalence of debilitating conditions
	3 × 500 mg metformin for 16 weeks	Non-diabetic pre-frail elderly patients	Human	Laksmi et al. (2017)	2017	Improved usual gait speed, but did not significantly improve grip strength and muscle growth inhibitor serum levels
	Starting dose of 500 mg daily and goal dose of 1,000 mg twice daily	Older adults with prediabetes aged more than 65 years	Human	Espinoza et al. (2020)	2020	Ongoing

### Osteoporosis

Bone is a dynamic tissue that is constantly metabolized in the body, maintaining structural integrity and mineralization balance through continuous destruction and regeneration. The bone content of the human body decreases significantly from roughly age 50 onwards. Osteoporosis has become an important challenge affecting the health of the elderly, especially postmenopausal women. Metformin plays an important role in bone homeostasis by inhibiting excessive oxidation. The *in vitro* study revealed that metformin (0.05–0.3 mM) could protect mitochondrial function and antagonize oxidative stress damage by upregulating SIRT3 expression, and ultimately alleviate H_2_O_2_ (0.2 mM)-induced apoptosis in osteoblasts (Yang et al., 2021). Similarly, another study found that metformin (50 mM) promoted cell differentiation and new bone formation in bone mesenchymal stem cells by a mechanism related to the regulation of autophagy, proliferation, and oxidative stress by metformin (Lin et al., 2020) *In vivo* experiments, oral metformin promoted the release of osteoprotegerin (OPG) in osteoblasts, thereby inhibiting the differentiation of macrophages to osteoclasts and reducing bone loss in the de-ovalized osteoporotic rat model (Mai et al., 2011). In a glucocorticoid-induced osteoporosis model, oral metformin (200 mg/kg/day) had a dual protective effect, i.e., it both inhibited bone resorption and promoted bone formation in trabeculae (Zhao et al., 2019), Another study identified a similar osteoprotective effect of metformin in ketogenic diet-induced osteoporosis mice (Liu et al., 2019). In addition, metformin was able to prevent osteonecrosis, and the area of osteonecrosis in the jaws of rats given metformin was significantly reduced compared to the control group (Nakagawa et al., 2021). Besides preclinical studies, a study discovered that metformin use in patients with type 2 diabetes reduced the risk of osteoporosis/vertebral fracture by 30–40% (Tseng, 2021). This also supported the emergence of metformin as a first-line drug for the treatment of type 2 diabetes. The bone-enhancing effects of metformin could reduce the risk of osteoporosis and fractures in diabetic patients (Kanazawa, 2009). In the Diabetes Prevention Program study, femoral neck bone mineral density was higher in the metformin group compared to placebo, suggesting that metformin may have a potentially positive effect on bone (Schwartz et al., 2021). Similarly, another study identified a small but positive effect of metformin treatment on the mineral content and density of peripheral bone (Nordklint et al., 2021). Notably, some researchers proposed that metformin administration was associated with a decreased risk of osteoporosis in adult women, regardless of whether they had type 2 diabetes or were obese (Blümel et al., 2020).

### Sarcopenia

Sarcopenia is usually defined as the loss of skeletal muscle mass and loss of function associated with aging. Skeletal muscle mass decreases with age, with approximately 30–50% loss of muscle mass by the time a person reaches 60 years of age (Ramírezgarcía et al., 2017). A meta-analysis enrolling 16,800 patients with type 2 diabetes suggested that metformin use was a protective factor for the development of sarcopenia (Ai et al., 2021). Metformin could enhance skeletal muscle function by promoting skeletal muscle differentiation and myotubular maturation (Senesi et al., 2016). However, the effect of metformin on muscle was still controversial, metformin (2 mM *in vitro* and 250 mg/kg *in vivo*) regulated muscle growth inhibitor through the AMPK-FoxO3a-HDAC6 axis and eventually induced muscle atrophy (Kang et al., 2021). Analysis of this controversy may be influenced by age and metabolic factors in rats, and long-term metformin administration leads to a decrease in androgen and estrogen levels in the body (Campagnoli et al., 2013). Hormone levels and changes in the metabolic environment *in vivo* may lead to differences in the regulation of muscle mass and function in rats of different ages. Researchers defined 12-months rats as “adult”, 18-month as “old”, adult rats administered metformin did show a decrease in whole-body lean tissue mass and no significant change in body fat content compared to controls, but older rats (from 18 to 24 months) administered metformin showed an increase in muscle and bone (Hernández-Álvarez et al., 2019). Many studies have found that metformin combined with exercise therapy can inhibit muscle mass loss and loss of function (Hernández-Álvarez et al., 2019; Hernández-Arciga et al., 2020; Toledo-Pérez et al., 2021). But a randomized controlled trial that included healthy older adults found that progressive resistance exercise training (PRT) (14 weeks) effectively counteracted skeletal muscle atrophy and induced increases in muscle mass and strength. However, the combination of metformin inhibited these benefits (Walton et al., 2019). In conclusion, the effect of metformin on skeletal muscle strengthening or atrophy remains to be explored. More experiments are probably necessary to investigate the relationship between metformin administration and skeletal muscle function.

### Degenerative Osteoarthrosis

Osteoarthritis (OA) is usually defined as a degenerative joint disease characterized by articular cartilage damage and reactive bone proliferation at the joint edges and under the cartilage. Notably, osteoarthritis is a disease that is closely associated with advancing age. Epidemiological studies predict that the prevalence of osteoarthritis in the population over 45 years of age will increase to 29.5% by 2032 (Hunter and Bierma-Zeinstra, 2019). Among the population suffering from osteoarthritis, the percentage of diabetics is as high as 54% (Barbour et al., 2017), this may be due to higher blood glucose levels and the accumulation of advanced AGEs that can accelerate the development of osteoarthritis (Lu et al., 2018).Chondrocyte senescence degeneration functions in the progression of osteoarthritis (Rim et al., 2020). Metformin treatment can activate the AMPK/mTOR signaling pathway to inhibit cartilage degeneration and aging, thus contributing to the treatment of OA (Feng et al., 2020). Li et al. conducted an in-depth study on the relationship between metformin and osteoarthritis. In the medial meniscus instability surgery-induced osteoarthritis model in mice, feeding metformin significantly inhibited articular cartilage degeneration, synovial tissue proliferation, and bone flab formation, while there was no similar effect in AMPKα1^−/−^ mice. Metformin not only exerts a chondroprotective effect by activating AMPK but also reduces pain sensitivity in OA patients. Researchers also conducted experiments in large animals, rhesus monkeys, and showed that metformin reduced cartilage damage and significantly increased the duration of standing and walking in rhesus monkeys (Li et al., 2020). Metformin has the potential to be an effective agent in slowing the progression of osteoarthritis. The age-related decrease in chondrocyte numbers and limited extracellular matrix production are important factors in the progression of osteoarthritic disease. Stem cell therapy has also attracted increasing interest from researchers (Menarim et al., 2019; Tang et al., 2021). A recent study investigated the ability of intra-articular injection of mesenchymal stromal cells to significantly increase cartilage thickness in an animal model (rabbits) (Köhnke et al., 2021). Mesenchymal stem cell therapy can not only improve the ability of chondrocytes to migrate from healthy to damaged tissues (Hopper et al., 2015), but also secrete cartilage repair-related proteins and trophic factors (Tang et al., 2021), which are essential for cartilage repair. Interestingly, metformin was found to enhance the immunomodulatory capacity of MSCs, resulting in increased analgesic activity and chondroprotective effects (Park et al., 2019). Additionally, the beneficial effects of metformin in osteoarthritis are supported by results from human studies. Results from a randomized, double-blind trial suggested that metformin could reduce levels of inflammatory mediators and enhance the therapeutic effect of NSAIDs alone (Mohammed et al., 2014). A retrospective cohort study in Taiwan investigated diabetic patients with osteoarthritis and suggested a decreased rate of surgery for joint replacement in patients who received combined metformin and Cox-2 inhibitors compared to Cox-2 inhibitors alone (Lu et al., 2018). Similarly, another prospective cohort study indicated that treatment with metformin resulted in a reduced rate of knee cartilage volume loss and a decreased risk of total knee arthroplasty (Yuanyuan Wang et al., 2019). However, there remains a dearth of high-quality randomized controlled trials evaluating the relationship between metformin and osteoarthritis, and whether metformin is indicated for non-diabetic patients with osteoarthritis deserves further investigation.

### Frailty

Frailty is generally defined as a non-specific state of aging-related decline in the function of various organ systems, increased vulnerability of the body, and diminished resistance to stressors. Compared to healthy individuals, frail older adults are more likely to experience falls, fractures, hospital admissions, complications, disability, and even death (Dent et al., 2019; Hale et al., 2019). However, it is worth noting that debilitation is dynamic and perhaps preventable. Patients with diabetes appear to be at higher risk for frailty, which may be attributed to the effects of hyperglycemia and insulin resistance on multiple systems, including the musculoskeletal system, the vascular endothelial system, and the endocrine system (Assar et al., 2019). Metformin is a highly effective hypoglycemic agent with anti-aging, anti-inflammatory, and anti-oxidative stress effects, and some researchers have suggested that it could be a potential preventative drug for frailty (Espinoza et al., 2019). A cross-sectional study investigating the use of metformin and the occurrence of frailty in veterans with diabetes has suggested that metformin exposure is associated with a decreased risk of frailty (Baskaran et al., 2020). Similarly, another study has revealed that the administration of metformin is a protective factor against the development of frailty syndrome in diabetic patients (Sumantri et al., 2014). However, there are few studies in this area and the results are inconsistent. One study with a follow-up period of 8–10 years has concluded that early exposure to lifestyle enhancement in people at risk for diabetes reduces the prevalence of frailty in later life, while metformin did not have a similar effect (Hazuda et al., 2021). The reason for the inconsistent analysis of the findings may be that the participants of the study are at risk for diabetes with an average age of 50 years, rather than true diabetics. Furthermore, the investigators have followed the occurrence of frailty in older adults in a prospective manner, which seems more convincing. In a study of non-prediabetic pre-frail elderly patients, it was found that gait speed was significantly higher in older adults taking metformin compared to placebo, while grip strength and muscle growth inhibitor serum levels did not change noticeably. However, the small sample size and large dropout rate were limitations of the trial (Laksmi et al., 2017). Espinoza et al. have designed a randomized double-blind controlled trial that proposes to incorporate elders (age ≥65 years) with prodromal diabetes and randomize them to the metformin and placebo groups. The trial is still ongoing to investigate whether metformin treatment will prevent or delay the onset of frailty by following up for 2 years (Espinoza et al., 2020). Frailty is a multidimensional state of diminished ability to maintain homeostasis in the internal environment and has a high prevalence in the elderly population. Epidemiological studies suggest that the prevalence of frailty in the community elderly population ranges from 14.9 to 31.9% (Ziller et al., 2020). Frailty can occur in both diabetic patients, people at risk for diabetes, and healthy older adults. Therefore, more large-scale, high-quality trials will be needed in the future to explore the correlation between metformin treatment and the occurrence of frailty syndrome in people with or without diabetes.

## The Side Effects of Metformin

Research has revealed that metformin has potential side effects. Some individuals experience transient gastrointestinal adverse reactions after taking metformin, including abdominal discomfort, diarrhea, nausea, vomiting, bloating, and dyspepsia (Wang and Hoyte, 2019; Calkin et al., 2022). In addition, metformin may increase lactate levels in mice and humans (Huang et al., 2017; Soukas et al., 2019), but these appear to be clinically insignificant and rare. In cases of renal insufficiency, drug accumulation may increase the risk of lactic acidosis, and patients can become dizzy, fatigued, or have gastrointestinal discomfort (Shoshan-Barmatz et al., 2021). Support has been provided for the fact that it also rarely occurs in individuals with severe renal insufficiency (Chu et al., 2020). Another side-effect of metformin is that it can cause vitamin B12 deficiency (Niafar et al., 2015; Wang and Hoyte, 2019). Lower vitamin B12 levels are associated with cardiovascular disease, neuropathy, and anemia (Zhang et al., 2017; Polat et al., 2022), but available studies suggest that metformin-induced vitamin B12 deficiency is usually not severe. Whether metformin will cause other adverse effects when taken long-term in a larger population remains unknown. Special attention should be paid to people with gastrointestinal discomfort, vital organ insufficiency, acute and chronic acidosis, and vitamin B12 deficiency.

## Conclusion and Perspectives

In summary, metformin, as a promising anti-aging drug, can play a protective effect in age-related diseases of the locomotor system. It can regulate bone, muscle, and joint functions and slow down the progression of osteoporosis, sarcopenia, and osteoarthritis. Metformin regulates intracellular signaling pathways through activation of AMPK or other pathways to exert protective effects such as anti-inflammatory, analgesic, and antagonistic oxidative stress. However, there are relatively few studies on metformin and the aging of the musculoskeletal system, and most of them are limited to animal models. Fewer studies have been conducted in people at high risk for diabetes or in healthy older adults. It is probably attributed to ethical constraints that many experiments cannot be conducted on healthy elderly people. However, studies in healthy individuals are necessary to understand whether metformin has an intrinsic role in regulating aging in addition to controlling diabetes. Future more convincing and larger studies, as well as studies in non-diabetic populations, are required to explore the anti-aging effects of metformin. Healthy elderly people, diabetic patients, and obese people will experience a decline in motor function or motor system disorders with increasing age. Metformin application may vary in individuals with different metabolic states, and the applicability, as well as the effectiveness of metformin, still requires further confirmation in high-quality, large-scale studies. Aging is a process that every living organism has to face, but how to slow down aging, reduce suffering and prevent aging-related diseases is a subject that needs to be continuously explored.
